# Transmission Clusters, Predominantly Associated With Men Who Have Sex With Men, Play a Main Role in the Propagation of HIV-1 in Northern Spain (2013–2018)

**DOI:** 10.3389/fmicb.2022.782609

**Published:** 2022-03-31

**Authors:** Horacio Gil, Elena Delgado, Sonia Benito, Leonidas Georgalis, Vanessa Montero, Mónica Sánchez, Javier E. Cañada-García, Elena García-Bodas, Asunción Díaz, Michael M. Thomson, The Members of the Spanish Group for the Study of New HIV Diagnoses

**Affiliations:** ^1^HIV Biology and Variability Unit, Centro Nacional de Microbiología, Instituto de Salud Carlos III, Madrid, Spain; ^2^HIV Surveillance and Behavioral Monitoring Unit, Centro Nacional de Epidemiología, Instituto de Salud Carlos III, Madrid, Spain

**Keywords:** Spain, HIV-1, transmission clusters, molecular epidemiology, men who have sex with men, migrants

## Abstract

Viruses of HIV-1-infected individuals whose transmission is related group phylogenetically in transmission clusters (TCs). The study of the phylogenetic relations of these viruses and the factors associated with these individuals is essential to analyze the HIV-1 epidemic. In this study, we examine the role of TCs in the epidemiology of HIV-1 infection in Galicia and the Basque County, two regions of northern Spain. A total of 1,158 HIV-1-infected patients from both regions with new diagnoses (NDs) in 2013–2018 were included in the study. Partial HIV-1 *pol* sequences were analyzed phylogenetically by approximately maximum-likelihood with FastTree 2. In this analysis, 10,687 additional sequences from samples from HIV-1-infected individuals collected in Spain in 1999–2019 were also included to assign TC membership and to determine TCs’ sizes. TCs were defined as those which included viruses from ≥4 individuals, at least 50% of them Spaniards, and with ≥0.95 Shimodaira-Hasegawa-like node support in the phylogenetic tree. Factors associated to TCs were evaluated using odds ratios (OR) and their 95% CI. Fifty-one percent of NDs grouped in 162 TCs. Male patients (OR: 2.6; 95% CI: 1.5–4.7) and men having sex with men (MSM; OR: 2.1; 95% CI: 1.4–3.2) had higher odds of belonging to a TC compared to female and heterosexual patients, respectively. Individuals from Latin America (OR: 0.3; 95% CI: 0.2–0.4), North Africa (OR: 0.4; 95% CI: 0.2–1.0), and especially Sub-Saharan Africa (OR: 0.02; 95% CI: 0.003–0.2) were inversely associated to belonging to TCs compared to native Spaniards. Our results show that TCs are important components of the HIV-1 epidemics in the two Spanish regions studied, where transmission between MSM is predominant. The majority of migrants were infected with viruses not belonging to TCs that expand in Spain. Molecular epidemiology is essential to identify local peculiarities of HIV-1 propagation. The early detection of TCs and prevention of their expansion, implementing effective control measures, could reduce HIV-1 infections.

## Introduction

The HIV-1 epidemic is still one of the major public health problems in Spain. Around 3,500–4,000 new diagnoses (NDs) of HIV-1 infection are reported every year, with an estimated incidence of NDs of 7.5 per 100,000 population in 2019 (HIV STI and Hepatitis Surveillance Unit, 2020). A decreasing trend in the incidence of NDs has been observed since 2010, although it is still higher than the average rate found in the EU/EEA (European Centre for Disease Prevention and Control and WHO Regional Office for Europe, 2020). In 2019, most (56%) of the NDs were diagnosed among men who had sex with men (MSM) and 36% of all reported NDs were in people born outside of Spain (HIV STI and Hepatitis Surveillance Unit, 2020).

Molecular epidemiology is an important tool for describing the HIV-1 epidemic (Brenner et al., 2013; Paraskevis et al., 2016; Wertheim et al., 2017; Board et al., 2020). Individuals whose transmission is related group phylogenetically in clades named transmission clusters (TCs). Due to the high genetic variability of HIV-1, phylogenetic analysis allows to reconstruct transmission events through the identified TCs and infer the history of the HIV-1 epidemic (Hué et al., 2004, 2005).

National databases of protease and reverse transcriptase (Pr-RT) sequences, primarily obtained for antiretroviral drug resistance testing, contain valuable data about HIV-1 expansion and have been used in molecular epidemiology studies (Ragonnet-Cronin et al., 2016a; Parczewski et al., 2017; Oster et al., 2018; Petersen et al., 2018; Verhofstede et al., 2018; Pineda-Peña et al., 2019; Fabeni et al., 2021). The phylogenetic analyses performed in these studies combined with clinical and epidemiological data of the patients provide relevant public health information for the implementation of control measures and for monitoring the HIV-1 epidemic (Brenner et al., 2013; Vasylyeva et al., 2016, 2020; Paraskevis et al., 2019; Board et al., 2020; Campbell et al., 2020).

Spanish clinical guidelines recommend to perform a genotypic drug resistance test before starting antiretroviral therapy (ART) in all HIV-1-diagnosed patients and in ART-failing cases [AIDS study group (GESIDA) of the Spanish Society of Infectious Diseases and Clinical Microbiology and the National AIDS Plan, 2020]. Pr-RT sequences obtained for these tests have been analyzed in different molecular epidemiology studies, describing the genetic features of the HIV-1 epidemic in different regions of Spain (Thomson et al., 2001; Holguín et al., 2007; Cuevas et al., 2009a,b; González-Alba et al., 2011; Yebra et al., 2013; Pérez-Parra et al., 2015, 2016; Patiño-Galindo et al., 2016, 2017b; González-Domenech et al., 2020). The phylogenetic studies have also allowed the identification of large TCs among MSM which are actively growing in Spain (Delgado et al., 2015, 2019; Patiño-Galindo et al., 2017a; González-Domenech et al., 2018), as well as transmitted drug resistance mutations which are spreading in TCs (Cuevas et al., 2009b; Vega et al., 2015; Viciana et al., 2016; González-Domenech et al., 2018, 2020), highlighting the value of molecular epidemiology as a tool for HIV surveillance.

The Basque Country and Galicia are two regions located in northern Spain, comparable in terms of population and HIV-1 diagnosis rates. During 2013–2018, the mean resident population and rates of new HIV diagnoses were estimated in 2,2 million and 6,9 per 100,000 inhabitants for the Basque Country and 2,7 million and 5,6 per 100,000 inhabitants for Galicia (HIV STI and Hepatitis Surveillance Unit, 2020; Instituto Nacional de Estadistica, 2021). In this study, we analyzed the frequency of grouping in TCs among patients diagnosed in 2013–2018 in these northern Spanish regions, identifying the different features associated with TCs in the HIV-1 epidemics in these regions.

## Materials and Methods

### Patients

Samples from individuals newly diagnosed of HIV-1 infection in the northern Spanish regions of Galicia and Basque Country during 2013–2018, sent to the HIV Biology and Variability Unit, Centro Nacional de Microbiología, Instituto de Salud Carlos III, were included in the study.

The representativeness of the NDs of our cohort was estimated comparing them to the NDs reported to the Spanish information system on new HIV diagnoses (SINIVIH by its Spanish acronym; HIV STI and Hepatitis Surveillance Unit, 2020), from the same period and studied regions. As patients in both databases cannot be linked, gender, age group, transmission route and region of birth frequencies were compared between both groups of individuals using the Chi-squared test.

The use of anonymized, de-identified clinical/demographic and sequence data was reviewed and approved under an exempt protocol by the Bioethics and Animal Well-being Committee of Instituto de Salud Carlos III, with report numbers CEI PI 38_2016-v3 (dated 20 June 2016) and CEI PI 31_2019-v5 (dated 6 November 2019). This study did not require written informed consent by study participants, except for those participants who required additional samples different from the ones obtained in the routine clinical practice.

### Nucleic Acid Extraction, Amplification, and Sequencing

Nucleic acid was extracted from plasma or whole blood samples. RNA was extracted from 1 ml plasma using NUCLISENS® easyMAG® (BioMérieux, Marcy l’Etoile, France) and DNA was extracted from 200 μl whole blood using QIAamp® DNA DSP blood mini kit (Qiagen, Hilden, Germany), following the manufacturer’s instructions. A Pr-RT fragment of *pol* (HXB2 positions 2253–3629) was amplified by RT-PCR followed by nested PCR from RNA or by nested PCR from DNA. Reagents, PCR thermal profiles, and primers are described in Supplementary Tables 1, 2.

Population sequencing was performed with ABI Prism BigDye Terminator Cycle Sequencing kit and ABI 3730 XL sequencer (Applied Biosystems, Foster City, CA, United States) in the Genomic Unit of Instituto de Salud Carlos III. Sequences were assembled with SeqMan Pro v.12.2.1 (DNA STAR Lasergene, Madison, WI, United States) and edited with BioEdit v.7.2.5 (Hall, 1999).

### Phylogenetic Analyses and Transmission Cluster Identification

Partial *pol* sequences from the patients included in this study and sequences obtained by us from another 10,687 HIV-1-infected individuals attended in clinical centers from Spain whose samples were collected in the period 1999–2019 were included in the analyses. A single sequence per patient was used in the phylogenetic analysis. In cases where several sequences were available for one patient, that closer to the date of HIV-1 diagnosis was selected.

Reference sequences from the different subtypes and CRFs retrieved from the Los Alamos HIV Sequence Database^1^ were included for the phylogenetic and bootscanning analysis. In addition, we conducted BLAST (Altschul et al., 1990) searches of each of the sequences obtained by us against the Los Alamos database, including in the phylogenetic analysis up to 10 most similar sequences from each search.

Sequences were analyzed phylogenetically by an approximately maximum-likelihood method using FastTree2 (Price et al., 2010), running it in a local desktop computer. In these analyses, the general time reversible model of nucleotide substitution with CAT approximation to account for among-site heterogeneity in substitution rates was used, and the reliability of nodes was assessed with Shimodaira–Hasegawa (SH)-like local support values (Shimodaira and Hasegawa, 1999; Guindon et al., 2010). Classification of sequences in subtypes and recombinant forms was based on clustering with clade references in approximately maximum-likelihood trees. Sequences suspected of intersubtype recombination were subsequently analyzed by bootscanning with SimPlot v3.5 (Lole et al., 1999).

Transmission clusters were defined as those comprising viruses from four or more individuals, at least 50% of them Spanish, and whose sequences grouped in the phylogenetic tree with a SH-like node support value ≥ 0.95. This limit in the percentage of migrants included in TCs was used to minimize the effect of viruses which are circulating in the countries of origin of the migrants, grouping phylogenetically in TCs in our sequence dataset due to the relatedness of the viruses of those countries, although they are not actually spreading in Spain.

### Statistical Analysis

To evaluate the association between variables, Chi-squared and Fisher’s exact tests were used, with associations being considered statistically significant at a value of *p* < 0.05. A multivariable logistic regression model was performed to identify factors associated to TCs. The model was adjusted by gender, age group, transmission route, country of origin of the patient, Spanish region of sample collection and HIV-1 genetic form. Associations were measured using the odds ratio (OR) and its 95% CI. Data analyses were performed using the STATA statistical software package Version 16 (Stata Corporation, College Station, TX, United States).

### Sequence Accession Numbers

To avoid the identification of transmission networks and the potential breech of patient confidentiality, only 19% of the sequences have been deposited in GenBank, including at least two sequences from each TC comprising five or more patients with new HIV-1 diagnoses in the period of study. The sequences are under the following accession numbers: KT276255, KT276260, KT276263-KT276264, KT276266-KT276267, KT276270-KT276271, KU685562-KU685565, KU685567, KU685569-KU685575, KU685577, KU685581-KU685582, KU685586, KU685588-KU685590, KU685592, KX534325, KX534329, KX534331, KY465968, KY496624, KY514084, KY989950, MF999250-MF999256, MF999258-MF999261, MK177721-MK177733, MK177735-MK177752, MK177754-MK177757, MK177761-MK177772, MK177783-MK17785, MK177787-MK177790, MK177792, MK177795-MK177796, MK177798-MK177799, MK177801-MK177803, MK177805-MK177807, MK298150, MT436242, MT436244, MT436246-MT436247, MT436249-MT436250, MW344920-MW344921, MW584217-MW584224, OK011532, OK011542, OK011545-OK011546, OK011549, OK148912, OK148914, OK148917-OK148919, OK148921, OK148941-OK148942, OK148953-OK148957, OK148961, OK148965, OK148968-OK148972, OK148974, OL982314-OL982315, and OM914651-OM914711.

## Results

### Study Population

A total of 1,158 HIV-1 NDs from Galicia and Basque Country diagnosed in 2013–2018 were included in the study (Table 1). The majority of the patients were male (82%), with MSM being the most common transmission route (46%). Heterosexuals represented 34% and 15% of the individuals were male whose transmission route was reported as sexual without specifying whether they were in the MSM or heterosexual category. Subtype B (63%) was the most frequent genetic form among the individuals included in the study, followed by unique recombinant forms (URFs; 8.2%), CRF02_AG (7.7%), and subtype F (7.6%; Table 1).

**Table 1:** Characteristics of the HIV-1 newly diagnosed individuals included in the study.

		BC^*^	GA^†^	All	
		*N* (%)	*N* (%)	*N* (%)	*p*-value
Gender	Male	626 (82)	306 (83)	932 (82)	0.71
	Female	133 (17)	65 (17)	198 (17)	
	Transexual	5 (0.65)	0 (0)	5 (0.44)	
	Unknown	12	11	23	
Transmission route	MSM^‡^	317 (47)	142 (44)	459 (46)	0.61
	Heterosexual	233 (34)	112 (35)	345 (34)	
	MNSST^§^	103 (15)	48 (15)	151 (15)	
	PWID^¶^	22 (3.2)	17 (5.3)	39 (3.9)	
	Other	6 (0.88)	3 (0.93)	9 (0.90)	
	Unknown	95	60	155	
Region of origin	Spain	477 (65)	268 (83)	745 (70)	<0.001
	Latin America	147 (20)	37 (11)	184 (17)	
	Sub-Saharan Africa	79 (11)	6 (1.9)	85 (8.0)	
	North Africa	14 (1.9)	1 (0.3)	15 (1.4)	
	Europe^∥^	16 (2.2)	12 (3.7)	28 (2.6)	
	Other	5 (0.68)	1 (0.31)	6 (0.56)	
	Unknown	38	57	95	
Genetic form	B	489 (63)	235 (62)	724 (63)	<0.001
	F	39 (5.0)	49 (13)	88 (7.6)	
	A	21 (2.7)	12 (3.1)	33 (2.9)	
	C	19 (2.5)	20 (5.2)	39 (3.4)	
	G	12 (1.6)	8 (2.1)	20 (1.7)	
	CRF02_AG	77 (9.9)	12 (3.1)	89 (7.7)	
	CRF_BF	20 (2.6)	8 (2.1)	28 (2.4)	
	URF	65 (8.4)	30 (7.9)	95 (8.2)	
	Other	34 (4.4)	8 (2.1)	42 (3.6)	
Total		776	382	1158	

* *BC, Basque Country*.

† *GA, Galicia*.

‡ *MSM, men who have sex with men*.

§ *MNSST, men who have non-specified sexual transmission*.

¶ *PWID, person who injects drugs*.

∥ *Other than Spain*.

Seventy percent of the patients were native Spanish (Table 1), followed by Latin Americans (17%) and Sub-Saharan Africans (8.0%). There were important differences in gender and transmission route proportions between these two migrant populations, with female [52% (44/85) vs. 26% (37/180)] and heterosexual transmission [88% (65/74) vs. 33% (54/165)] being higher in Sub-Saharan Africans than in Latin Americans.

The distribution of patients by gender and transmission route was similar in both studied regions (Table 1), although the frequency of people who inject drugs (PWID) was slightly higher in Galicia (5.3 vs. 3.2%), but the difference was not statistically significant (*p* = 0.13). The percentage of patients of non-Spanish origin was 35% in the Basque Country (13% corresponding to Africans), a proportion which is statistically higher (*p* < 0.001) compared to the 17% found in Galicia (2.2% Africans). Statistical differences in the distribution of genetic forms were also observed between both regions (*p* < 0.001), with the frequencies of subtype F (13%) and CRF02_AG (9.9%) being higher in Galicia and the Basque Country, respectively (Table 1).

### Representativeness of the Patients Included in the Study

Our cohort of newly diagnosed HIV-1-infected patients represents 64% of the notified cases in 2013–2018 (Supplementary Table 3), assuming that all NDs included in our study have been reported to the SINIVIH. This percentage was higher in the Basque Country, where it reached 86%, than in Galicia, where it was 42% of the notified cases. No statistical differences were found between both patient groups regarding the distribution of individuals by gender, transmission route, region of origin, or age group (Supplementary Table 3). A map with the studied regions, with the numbers of sequences included in our dataset and the representativeness in each region of the NDs analyzed in this study is shown in Supplementary Figure 1.

### TCs Identified in the Study

Fifty-one percent (594/1158) of the individuals belonged to 162 different TCs. Regarding regions, 45% (351/776) of the patients were included in 98 TCs in the Basque Country and 64% (243/382) belonged to 82 TCs in Galicia (Table 2). Eighteen (11%) TCs comprised patients from both regions. The sizes of the TCs ranged from 4 to 205 patients. MSM was the main transmission route associated with 59% of the TCs (Table 3). Transmission routes other than MSM were more frequently associated with TCs in Galicia (44%) than in the Basque County (35%; Table 2), although the difference was not statistically significant (*p* = 0.207).

**Table 2:** Characteristic of the identified transmission clusters (TCs) of the study.

		Basque Country		Galicia		Both regions	
		*N*	%	*N*	%	*N*	%
Transmission route^*^	MSM^†^	64	65	46	56	95	59
	Heterosexual	10	10	14	17	24	15
	Sexual^‡^	16	16	10	12	23	14
	PWID^§^	8	8.2	12	15	20	12
Total		98	100	82	100	162	100

* *Main mode of HIV transmission among individuals in the TC*.

† *MSM, men who have sex with men*.

‡ *There is not predominance of MSM or heterosexual transmission*.

§ *PWID, persons who inject drugs*.

**Table 3:** Characteristics of the transmission clusters comprising ≥30 patients in the Basque Country and Galicia (2013–2018).

Cluster	Genetic form	No. patients	Regional distribution						% Spanish	% Male	% Transmission route^‡^ (MSM/HT/MNSST/PWID)	Patients (2013–18)^§^		Period^¶^
			BC^*^		GA^†^		Others							
			*N*	%	*N*	%	*N*	%				*N*	%	
F1_1	F1	205	9	4.4	138	67	58	28	85	97	72/13/14/0	78	38	2000–2019
CRF02_1	CRF02_AG	83	34	41	3	3.6	46	55	79	92	64/22/12/2	44	53	2008–2019
B50	B	74	15	20	3	4.1	56	76	85	100	69/9/22/0	34	46	2007–2019
A1_1	A1	63	8	13	24	38	31	49	71	95	77/12/11/0	48	76	2006–2019
B70	B	61	56	92			5	8.2	88	98	64/9/27/0	44	72	2008–2019
B08	B	60	46	77	6	10	8	13	92	98	74/6/19/2	13	22	2006–2019
B09	B	57	55	97			2	3.5	88	98	62/15/21/2	10	18	1991–2019
B13	B	55			54	98	1	1.8	89	94	62/19/19/0	16	29	2004–2017
B31	B	49	19	39	2	4.1	28	57	77	98	73/7/20/0	23	47	2001–2019
B07	B	38	36	95			2	5.3	91	95	56/12/29/3	3	7.9	2000–2019
B10	B	36	29	81			7	19	90	97	67/12/18/3	7	19	1991–2019
F1_3	F1	36	29	81			7	19	76	100	61/11/29/0	34	94	2014–2018
B12	B	35	25	71	1	2.9	9	26	63	100	67/7/26/0	11	31	2009–2019
BG_2	URF_BG	33			33	100			85	80	0/45/3/52	2	6.1	1994–2019
B05	B	31			31	100			93	94	56/26/19/0	10	32	2003–2019

* *BC, Basque Country*.

† *GA, Galicia*.

‡ *Percentages were calculated from the total of patients with known transmission route*.

*MSM, men who have sex with men; HT, heterosexual; MNSST, men who have non-specified sexual transmission; and PWID, people who inject drugs*.

§ *Newly-diagnosed HIV-1 patients during 2013–2018*.

¶ *Year of HIV-1 diagnosis of the patients in the TC*.

Non-subtype B genetic forms were found in 33 (21%) of the 162 TCs identified in the study. Sixteen of them derived from genetic forms circulating in Latin America (BF1 recombinants, Brazilian subtype C and F1 variants, CRF19_cpx and CRF20_BG) and 10 from genetic forms circulating in Africa (CRF02_AG, CRF02/A3, CRF02/G, CRF06_cpx and subtype C strains of African origin). Twenty-nine percent (138/474) of the Spaniards belonging to TCs were members of non-subtype B TCs.

A total of 15 large TCs (here defined as those with ≥30 individuals) were identified in the study (Table 3), 10 of them of subtype B. MSM was the associated transmission route in 14 (93%) of them. Seventy-six percent (67/88) of subtype F infections belonged to the MSM-associated TCs F1_1 and F1_3. Most of these large TCs have spread mainly in a specific region. Thus, TCs B07, B08, B09, B10, B12, B70, and F1_3 were found mainly in the Basque Country, while TCs F1_1, B13, BG_2, and B05 were spread mainly in Galicia (Table 3). In addition, A1_1, CRF02_1, B50 and B31 have a global expansion in Spain, with a high number of patients outside of both studied regions. Four TCs had greater than 50% increase in NDs during the period 2013–2018: F1_3 (94%), A1_1 (76%), B70 (72%), and CRF02_1 (53%; Table 3).

A phylogenetic tree which includes all the TCs comprising five or more patients newly diagnosed in the studied period in Galicia and Basque Country is shown in Supplementary Figure 2.

### Factors Associated With TCs

The percentage of patients in TCs was higher in males (57%) than in females (22%), and in MSM (67%) than in heterosexuals (31%). This percentage was also higher in Spaniards (64%) than in Latin Americans (35%) or Sub-Saharan Africans (1.2%), and in individuals infected with viruses of subtypes F (76%) or B (58%) than in those infected with CRF02_AG viruses (17%). The different distribution of TCs according to gender, mode of HIV-1 transmission, country of origin of the patient, and HIV-1 genetic form were statistically significant (Table 4). The age group was also associated to TCs (*p* = 0.036). The age range of 20–29 years showed an increased percentage of patients in TCs (59%), which was especially high in Galicia (81%, *p* = 0.003) but was not statistical significant in the Basque Country, and no statistical differences were found in the distribution of main variables.

**Table 4:** Characteristics of the new HIV diagnoses included in TCs (2013–2018).

		**Basque Country**					Galicia					**Both regions**				
**Variables**	**Categories**	**No**		**Yes**		***P*** **-*value***	**No**		**Yes**		***P*** **-*value***	**No**		**Yes**		***P*** **-*value***
		*N* ^*^	**%**	***N***	**%**		***N***	**%**	***N***	**%**		***N***	**%**	***N***	**%**	
Gender (*n* = 1135, no data = 23)	Female	115	86	18	14	<0.001	40	62	25	38	<0.001	155	78	43	22	<0.001
	Male	303	48	323	52		95	31	211	69		398	43	534	57	
	Transexual	3	60	2	40		0	0	0	0		3	60	2	40	
Transmission route (*n* = 1003, no data = 155)	Heterosexual	178	76	55	24	<0.001	60	54	52	46	<0.001	238	69	107	31	<0.001
	MSM^†^	120	38	197	62		33	23	109	77		153	33	306	67	
	MNSST^‡^	48	47	55	53		18	37	30	63		66	43	85	56	
	PWID^§^	15	68	7	32		8	47	9	53		23	59	16	41	
	Other	4	67	2	33		3	100	0	0		7	78	2	22	
Region of origin (*n* = 1063, no data = 95)	Spain	189	40	288	60	<0.001	82	31	186	69	0.001	271	37	474	64	<0.001
	Latin America	102	69	45	31		18	49	19	51		120	65	64	35	
	Sub-Saharan Africa	79	100	0	0		5	83	1	17		84	99	1	1.2	
	North Africa	9	64	5	36		1	100	0	0		10	67	5	33	
	Europe^¶^	11	69	5	31		7	58	5	42		18	64	10	36	
	Other	4	80	1	20		1	100	0	0		5	83	1	17	
Age group (*n* = 1133, no data = 25)	<20	10	53	9	47	0.44	3	50	3	50	0.003	13	52	12	48	0.036
	20–29	92	51	90	49		14	19	60	81		106	41	150	59	
	30–39	160	58	115	41		49	41	72	59		209	53	187	47	
	≥40	161	55	137	45		68	43	90	57		229	50	227	50	
Genetic form (*n* = 1158, no data = 0)	A	14	67	7	33	<0.001	3	25	9	75	0.002	17	52	16	48	<0.001
	B	224	46	265	54		83	35	152	65		307	42	417	58	
	C	11	58	8	42		8	40	12	60		19	49	20	51	
	F	10	26	29	74		11	23	38	78		21	24	67	76	
	G	10	83	2	17		5	63	3	37		15	75	5	25	
	CRF02_AG	63	82	14	18		11	92	1	8.3		74	83	15	17	
	CRF_BF	18	90	2	10		4	50	4	50		22	79	6	21	
	URF	47	72	18	28		10	33	20	67		57	60	38	40	
	Other	28	82	6	18		4	50	4	50		32	76	10	24	

* *N, number of patients*.

† *MSM, men who have sex with men*.

‡ *MNSST, men who have non-specified sexual transmission*.

§ *PWID, people who inject drugs*.

¶ *Spain not included*.

Along the studied period, there was no clear trend in the proportion of patients associated with TCs (Supplementary Figure 3). In Galicia, a decrease of notified HIV-1 NDs (Supplementary Figure 3, panel A) with a slight reduction of the number of TCs and an increase in the ratio of patients per TC (Supplementary Figure 3, panel D) was observed during the study period.

In the multivariate analysis, males (OR: 2.6; 95% CI: 1.5–4.7) and MSM (OR: 2.1; 95% CI: 1.4–3.2) had higher odds of belonging to a TC compared to females and heterosexuals, respectively (Table 5). Patients from Galicia (OR: 1.5; 95% CI: 1.1–2.2) and subtype F infections (OR: 2.5; 95% CI: 1.3–5.0) were also factors associated with TCs (Table 5).

**Table 5:** Factors associated with transmission clusters, univariate/multivariate analysis.

		**Univariate analysis**		**Multivariate analysis**	
**Variables**		**OR** ^*^	**95% CI** ^†^	**Adjusted OR**	**95% CI**
Gender	Female	Reference		Reference	
	Male	4.8	3.3–7.1	2.6	1.5–4.7
	Transexual	2.4	0.4–15	NA^‡^	NA
Transmission route	Heterosexual	Reference		Reference	
	MSM^§^	4.5	3.2-6.1	2.1	1.4–3.2
	MNNSST^¶^	2.9	1.9-4.3	1.4	0.8–2.3
	PWID^∥^	1.6	0.8-3.1	NA	NA
	Other	0.6	0.1–3.1	NA	NA
Region of origin	Spain	Reference		Reference	
	Latin America	0.3	0.2–0.4	0.3	0.2–0.4
	Sub-Saharan Africa	<0.01	<0.001–0.06	0.02	0.003–0.2
	North Africa	0.3	0.1–0.9	0.4	0.2–1.0
	Europe^ƪ^	0.3	0.1-0.7	0.3	0.03–3.5
	Other	0.1	0.1–1.0	0.4	0.1–1.6
Age group	<20	0.7	0.3–1.5	NA	NA
	20–29	Reference		Reference	
	30–39	0.6	0.5–0.9	0.6	0.4–0.9
	≥40	0.7	0.5–1.0	0.6	0.4–0.9
Spanish region	Basque Country	Reference		Reference	
	Galicia	2.1	1.6–2.7	1.5	1.1–2.2
Genetic form	A	0.7	0.3–1.4	NA	NA
	B	Reference		Reference	
	C	0.8	0.4–1.5	NA	NA
	F	2.4	1.4–3.9	2.5	1.3–5.0
	G	0.3	0.1–0.7	0.4	0.1–1.5
	CRF02_AG	0.2	0.1–0.3	0.6	0.3–1.2
	CRF_BF	0.2	0.1–0.5	0.1	0.03–0.4
	URF	0.5	0.3–0.8	0.8	0.4–1.4
	Other	0.4	0.3–0.7	0.3	0.2–0.8

* *OR, odds ratio*.

† *95% CI, 95% confidence interval*.

‡ *NA, not applicable*.

*Adjusted OR were not calculated for categories with *p* < 0.1 in the univariate analysis*.

§ *MSM, men having sex with men*.

¶ *MNSST, men who have non-specified sexual transmission*.

∥ *PWID, people who inject drugs*.

ƪ *Spain not included*.

Individuals from Latin America (OR: 0.3; 95% CI: 0.2–0.4), North Africa (OR: 0.4; 95% CI: 0.2–1.0) and especially from Sub-Saharan Africa (OR: 0.02; 95% CI: 0.003–0.2) were inversely associated to belonging to TCs compared to Spanish patients (Table 5). Similarly, patients over 29 years old (OR: 0.6; 95% CI: 0.4–0.9) and infections with a virus of a CRF_BF (OR: 0.1; 95% CI: 0.03–0.4) or of a genetic form classified in the “other” category (OR: 0.3; 95% CI: 0.2–0.8) had lower odds of belonging to TCs than patients in the age range of 20–29 years and with subtype B infections, respectively (Table 5). These associations, although with slightly lower OR, where also observed when the criterion on the country of origin of the patients was excluded from the TC definition (Supplementary Table 4).

## Discussion

The phylogenetic analysis of sequences from almost 12,000 HIV-1-infected patients from our database has allowed us to investigate the transmission events occurring during the 2013–2018 period in two regions of northern Spain, Galicia and Basque Country, identifying TCs and their associated factors. Our ND cohort had a good representativeness, corresponding to 64% of the NDs reported to the SINIVIH from these regions during that period, especially in the Basque Country where it reached 86%.

Our analysis has assigned 51% of the NDs from Galicia and Basque Country to 162 different TCs, indicating that TCs are playing an important role in the spread of HIV-1 infections in both regions. There is no consensus on the criteria for defining a TC, as these should depend on the aim of the study (Hassan et al., 2017). We considered only TCs including at least 50% Spaniards and at least four documented infections, because they may be more epidemiologically relevant to describe HIV-1 strains spreading in an area. Smaller clusters comprising two (“pairs”) or three (“triplets”) individuals are much more numerous than larger ones (Hughes et al., 2009; Brown et al., 2011; Yebra et al., 2013) and in some studies are distinguished from larger clusters (Hoenigl et al., 2016; Fabeni et al., 2020). They could represent sexual couples with or without a second relationship by one of the members of the couple, and their epidemiologic relevance is uncertain. The inclusion of these requirements in the TC definition has allowed us to better identify the structure and factors associated to HIV-1 strains whose transmission is established in Spain.

Transmission clusters were found associated with male gender and MSM. In fact, almost all the largest TCs (≥30 patients) identified in our study were associated with MSM. HIV-1 molecular epidemiological studies in different countries, independently of the methodology used, have found a similar association (Parczewski et al., 2017; Wertheim et al., 2017; Ramirez et al., 2021). Also, studies in other Spanish regions have found this association of TCs with MSM (Pérez-Parra et al., 2015; González-Domenech et al., 2020). These findings indicate that MSM TCs are playing a main role in spreading HIV-1 infections and are consistent with the fact that MSM is the main frequent mode of HIV-1 transmission in Spain since the early 2000s (Nuñez et al., 2018).

In Spain, 36% of HIV-1 infections diagnosed in 2019 were in migrants (HIV STI and Hepatitis Surveillance Unit, 2020). Migrants play an important role in HIV-1 epidemic spread, contributing to bridging between networks from different geographic areas (Grossman et al., 2015; Su et al., 2018; Grabowski et al., 2020). In this study, we have examined the frequency of membership in Spanish TCs of migrants from different geographic areas. We have found that Latin Americans and Africans showed lower odds of belonging to TCs than Spaniards. However, there were important differences between these migrant populations, as 35% of Latin Americans were included in TCs, compared to only 1.2% of Sub-Saharan Africans, suggesting that HIV-1 transmission between Latin Americans and Spaniards is relatively common, presumably derived from cultural and linguistic affinities (Osorno-González de León et al., 2021). Similarly to our results, a low percentage of Sub-Saharan Africans in TCs have been also reported in Italy (Fabeni et al., 2020), Belgium (Verhofstede et al., 2018), and Madrid, Spain (Yebra et al., 2013), while in Israel, cross-ethnic HIV-1 spread from Israeli-born individuals to Ethiopian-born immigrants was rare (Grossman et al., 2015). In our study, TCs are mainly MSM-driven. In contrast, we have found high frequencies of females (52%) and heterosexuals (88%) in Sub-Saharan Africans, which, in addition to the likely HIV-1 acquisition in their countries of origin, can explain the low proportion of individuals in this migrant population belonging to TCs. Indeed, we have observed that Sub-Saharan Africans are infected frequently with genetic forms circulating in Africa.

Determining the origin of HIV-1 infections in the migrant population would allow proper allocation of resources for preventive measures (Fakoya et al., 2015; Álvarez del Arco et al., 2017). Previous studies based on models using clinical and behavioral data estimated high levels of post-migration HIV acquisition in Europe (Desgrées-du-Loâ et al., 2015; Álvarez del Arco et al., 2017), up to 71% among migrants in Spain (Álvarez del Arco et al., 2017). This figure could be an overestimate due to biases in data collection and failure to consider infections acquired by migrants when visiting their countries, as migrants’ mobility is associated with increased risk for HIV acquisition (Fenton et al., 2001; Dias et al., 2020). Phylogenetic analyses can provide additional information on the geographical area of HIV-1 acquisition. In our study, the lower percentage of foreigners found in TCs could be due to infections with virus strains which are not circulating in Spain, suggestive of imported infections, which would be especially frequent in Sub-Saharan Africans. Further studies using a combined phylogenetic and epidemiological approach can address the accuracy of the origin of the HIV-1 infections in the migrant populations in Spain.

With regard to the role of immigration on the origin of HIV-1 TCs in Spain, it is also interesting to note that 21% TCs identified in this study are of non-subtype B genetic forms, with their ancestry showing some correlation with the predominant geographic origins of the immigrant population in Spain. Thus, the fact that 16 TCs derive from genetic forms circulating in Latin America and Africa may correlate to the high number of immigrants from these geographic areas living in Spain, representing around 36 and 18%, respectively, of the approximately 7.3 million immigrants (Instituto Nacional de Estadistica, 2021). Similarly, the expansion of a large F1 subsubtype cluster of Romanian origin (F1_3) in Spain (Delgado et al., 2019) may relate to the fact that among European immigrants in Spain, Romanians are the most numerous, representing around 8% of all immigrants (Instituto Nacional de Estadistica, 2021).

Subtype B was the most represented genetic form associated to TCs. Only subtype F infections (76% of NDs in TCs) showed a higher frequency than subtype B infections (56% of NDs in TCs) of belonging to TCs. This was mainly due to the presence of two large TCs of F1 subsubtype, designated F1_1 (205 individuals) and F1_3 (36 individuals), which have spread among MSM mainly in Galicia and Basque Country, respectively (Delgado et al., 2015, 2019). Interestingly, two other large non-subtype B TCs associated with MSM, of CRF02_AG and A1 subsubtype, are also present in these regions (Delgado et al., 2019). In Western Europe, the HIV-1 epidemic among MSM is dominated by subtype B. However, the epidemic among MSM in Spain is becoming increasingly diverse through the expansion of multiple non-subtype B TCs, comprising or related to viruses circulating in other countries (González-Domenech et al., 2018; Delgado et al., 2019). This phenomenon has been also documented in other European countries (Dauwe et al., 2015; Ragonnet-Cronin et al., 2016b; Verhofstede et al., 2018; Fabeni et al., 2019; Ramirez et al., 2021), where viruses introduced from abroad have expanded successfully among the MSM population.

In general, there was not a clear temporal trend in the proportion of patients belonging to TC, although in Galicia our data show an increase in the proportion of patients in TCs in the last years. However, trends are difficult to evaluate due to the presence of fast-growing TCs in some periods.

A different distribution of TCs is responsible for the spread of HIV-1 in Galicia and the Basque Country. Large MSM TCs have spread locally in both regions and the majority of the identified TCs were associated with this population group. However, we found a higher frequency of TCs associated with PWID or heterosexual transmission in Galicia. Galician patients have 1.5 higher odds of belonging to TCs than individuals from the Basque Country. This can be related to the low percentage of migrants in Galicia, who have a reduced association to TCs, and the high number of patients belonging to the large F1_1 TC, currently comprising 205 individuals, which has successfully spread in Galicia and other Spanish regions (Delgado et al., 2015), even in other European countries (Delgado et al., 2015; Vinken et al., 2019). We have found an association of young patients (age range 20–29 years) to TCs in Galicia, similarly to the findings in other studies (Brenner et al., 2017; Dennis et al., 2018; Verhofstede et al., 2018; Fabeni et al., 2019; Paraskevis et al., 2019; González-Domenech et al., 2020), but not in the Basque Country. Public health interventions for reducing high risk behavior and control measures in this age group in Galicia should be implemented to decrease HIV-1 transmissions in the region. Moreover, our results highlight that even regions with comparable demographic features can show a different HIV-1 epidemic pattern, likely due to specific social and cultural differences at the regional level.

In this study, we have shown that MSM TCs are a keystone of the HIV-1 epidemic at regional and probably also at the country level, according to the propagation of the large MSM TCs identified in this study in different regions of Spain. Consequently, reinforcement of public health measures for preventing HIV-1 infections in MSM, such as behavioral interventions to reduce risky practices, pre-exposure prophylaxis (Grant et al., 2010; McCormack et al., 2016; Riddell et al., 2018), and early diagnosis and treatment of HIV-1 infections (European Centre for Disease Prevention and Control, 2015; United Nations Population Fund Global Forum on MSM & HIV United Nations Development Programme World Health Organization United States Agency for International Development World Bank, 2015) are recommended.

Implementation of preventive public health measures targeting MSM could reduce indirectly the spread of HIV-1 in Spain, which is mainly MSM-driven, in other population groups. National surveillance data in the United States suggest that infections among heterosexual women predominantly originate from MSM (Oster et al., 2015). Moreover, the presence of female and self-declared heterosexual male individuals in MSM-associated TCs is frequent (Hué et al., 2014; Esbjörnsson et al., 2016; Ragonnet-Cronin et al., 2018; Verhofstede et al., 2018), as we have also observed in the large TCs in our study (Table 3). Mixed demographics in TCs allows the spread of HIV-1 infections between different population groups, as we have seen in the expansion of MSM-associated TCs to heterosexuals in Spain (Delgado et al., 2019), indicating the importance of monitoring the evolution of TCs for implementing proper control measures in each population group. Studies to assess the contribution of MSMs to HIV-1 transmission to other population groups are needed in Spain.

The high representativeness of our cohort, especially in the Basque Country, suggests that the largest and most spread TCs in these regions have been identified, which supports the conclusions of the study. However, our data may have some limitations as some of the NDs from Basque Country and Galicia during the studied period were not analyzed and not all the TCs propagating in Spain were detected in our phylogenetic analysis.

Our study has provided valuable data on the HIV-1 epidemic in Spain, identifying specific features at geographical and population levels for tailoring more specific public health interventions. The implementation of a molecular HIV-1 surveillance system to monitor the evolution of the epidemic in Spain could allow to promptly detect rapidly expanding TCs needing urgent investigation and the implementation of additional public health measures to prevent their spread (Centers for Disease Control and Prevention, 2018), as well as to readjust the control measures in place according to the evolution of the TCs.

## Members of the Spanish Group for the Study of New HIV Diagnoses

Basque Country: Hospital Universitario de Araba, Vitoria: Andrés Canut-Blasco, José Joaquín Portu, Carmen Gómez-González; Hospital Universitario de Basurto, Bilbao: Josefa Muñoz, Mª Carmen Nieto, María Zuriñe Zubero, Silvia Hernáez-Crespo, Estibaliz Ugalde; Hospital Universitario de Cruces, Barakaldo: Luis Elorduy, Elena Bereciartua, Leyre López Soria; Hospital de Galdakao: Mª José López de Goicoechea, José Mayo; Hospital Universitario Donostia: Gustavo Cilla, Julio Arrizabalaga, José Antonio Iribarren, Mª Jesús Bustinduy, Mª Julia Echevarría. Mª Yolanda Salicio, David Grandioso. Galicia: Área sanitaria de Ferrol: Ana Mariño, Patricia Ordóñez, Hortensia Álvarez, Nieves Valcarce; Complejo Hospitalario Universitario de A Coruña: Ángeles Cañizares, Mª Ángeles Castro. Hospital Universitario Lucus Augusti, Lugo: Ramón Rabuñal-Rey, María José García-País, Mª José Gude-González, Pilar Alonso-García, Antonio Moreno-Flores; Complejo Hospitalario Universitario de Ourense: Juan García Costa, Ricardo Fernández-Rodríguez, Raúl Rodríguez-Pérez, Jorge Guitián, María Dolores Díaz-López, María Genoveva Naval-Calviño; Complejo Hospitalario Universitario de Vigo: Celia Miralles, Antonio Ocampo, Sonia Pérez-Castro, Jorge Julio Cabrera; Complejo Hospitalario Universitario de Pontevedra: Julio Díz-Arén, Matilde Trigo, Mª Ángeles Pallarés. Navarra: Complejo Hospitalario de Navarra, Pamplona: Carmen Ezpeleta Baquedano, Carmen Martín Salas, Irati Arregui García, María Gracia Ruiz de Alda. Madrid: Centro Sanitario Sandoval, Madrid: Jorge del Romero, Carmen Rodríguez, Mar Vera, Óskar Ayerdi, Eva Orviz; Hospital Universitario de Fuenlabrada: María Isabel García-Arata, Santiago Prieto-Menchero; Hospital Clínico Universitario San Carlos, Madrid: Esther Culebras, Icíar Rodríguez-Avial; Hospital Universitario Fundación Jiménez Díaz, Madrid: Raquel Téllez-Pérez, Olalla Calabia-González, Alfonso Cabello-Úbeda, Miguel Górgolas Hernández-Mora; Hospital Universitario Severo Ochoa, Leganés: Sara María Quevedo, Lucía Puente, Manuel del Álamo; Hospital Fundación Alcorcón, Madrid: Carolina Campelo Gutiérrez, María José Goyanes Galán; Castilla y León: Hospital Clínico Universitario de Valladolid: Carmen Hinojosa, Carlos Dueñas, Begoña Monteagudo, Edita Sánchez; Hospital Río Hortega, Valladolid: Jessica Abadía, Belén Lorenzo Vidal; Hospital Virgen de la Concha, Zamora: Teresa Martín-Domínguez, Rosa Martínez-González. La Rioja: Hospital San Pedro, Logroño: José Ramón Blanco, Miriam Blasco. Aragón: Hospital Universitario Miguel Servet, Zaragoza: Ana María Martínez-Sapiña, Piedad Arazo. Castilla-La Mancha: Hospital Universitario de Guadalajara: Alejandro González Praetorius, Complejo Hospitalario de Toledo: César Gómez-Hernando, José Largo-Pau; Comunitat Valenciana: Hospital Universitari Sant Joan d’Alacant: Fernando Buñuel, Ana Infante.

## Data Availability Statement

The datasets presented in this study can be found in online repositories. The names of the repository/repositories and accession number(s) can be found at: https://www.ncbi.nlm.nih.gov/genbank/, KT276255, KT276260, KT276263-KT276264, KT276266-KT276267, KT276270-KT276271, KU685562-KU685565, KU685567, KU685569-KU685575, KU685577, KU685581-KU685582, KU685586, KU685588-KU685590, KU685592, KX534325, KX534329, KX534331, KY465968, KY496624, KY514084, KY989950, MF999250-MF999256, MF999258-MF999261, MK177721-MK177733, MK177735-MK177752, MK177754-MK177757, MK177761-MK177772, MK177783-MK17785, MK177787-MK177790, MK177792, MK177795-MK177796, MK177798-MK177799, MK177801-MK177803, MK177805-MK177807, MK298150, MT436242, MT436244, MT436246-MT436247, MT436249-MT436250, MW344920-MW344921, MW584217-MW584224, OK011532, OK011542, OK011545-OK011546, OK011549, OK148912, OK148914, OK148917-OK148919, OK148921, OK148941-OK148942, OK148953-OK148957, OK148961, OK148965, OK148968-OK148972, OK148974, OL982314-OL982315, and OM914651-OM914711.

## Ethics Statement

The studies involving human participants were reviewed and approved by Bioethics and Animal Well-being Committee of Instituto de Salud Carlos III. Written informed consent for participation was not required for this study in accordance with the national legislation and the institutional requirements.

## Author Contributions

HG, ED, and MT conceived the study and supervised the experimental work. ED, HG, MT, and JC-G processed sequences and performed phylogenetic analyses. HG and ED performed data curation. HG, AD, and LG performed statistical analyses. SB, MS, VM, and EG-B performed experimental work. The members of the Spanish Group for the Study of New HIV Diagnoses recruited patients and obtained epidemiological and clinical data. HG wrote the manuscript draft, with contributions to the text by AD, MT, and ED. All authors contributed to the article and approved the submitted version.

## Funding

This work was funded through Acción Estratégica en Salud Intramural (AESI), Instituto de Salud Carlos III, Project “Estudios sobre vigilancia epidemiológica molecular del VIH-1 en España,” PI16CIII/00033 and Project “Epidemiología molecular del VIH-1 en España y su utilidad para investigaciones biológicas y en vacunas“PI19CIII/0042; Red de Investigación en SIDA (RIS), Instituto de Salud Carlos III, Subdirección General de Evaluación y Fondo Europeo de Desarrollo Regional (FEDER), Plan Nacional ICDCI, project RD16ISCIII/0002/0004; and scientific agreements with Consellería de Sanidade, Government of Galicia (MVI 1004/16), and Osakidetza-Servicio Vasco de Salud, Government of Basque Country (MVI 1001/16).

## Conflict of Interest

The authors declare that the research was conducted in the absence of any commercial or financial relationships that could be construed as a potential conflict of interest.

## Publisher’s Note

All claims expressed in this article are solely those of the authors and do not necessarily represent those of their affiliated organizations, or those of the publisher, the editors and the reviewers. Any product that may be evaluated in this article, or claim that may be made by its manufacturer, is not guaranteed or endorsed by the publisher.

## Footnotes

^1^ https://www.hiv.lanl.gov/content/sequence/HIV/mainpage.html
