# Supplementary material for: Transmission Clusters, Predominantly Associated With Men Who Have Sex With Men, Play a Main Role in the Propagation of HIV-1 in Northern Spain (2013–2018)
Source: Front Microbiol. 2022 Mar 31;13:782609. doi: 10.3389/fmicb.2022.782609 (PMC9009226; doi:10.3389/fmicb.2022.782609)
Supplement: Supplementary file 1 [file Table_2.PDF]

Supplementary Table 1: Reagents and condition of the Pr-RT amplification.

Reagents	RT-PCR (for RNA)	1st PCR (for DNA)	Nested PCR (RNA and DNA)
OneStep RT-PCR (QIAGEN) enzyme	1 µl	--	--
GoTaq G2 Hot Start colorless mastermix (Promega)	--	12.5 µl	--
BioTaq DNA polymerase (Bioline)	--	--	0.75 U
RNAse inhibitor	5 U	--	-
$MgCl_2$	2.5 mM*	2mM†	1.8 mM
dNTPs	400 µM	200 µM†	200 µM
Each Primer	0,6 µM	0,4 µM	0,4 µM
Reaction volume	25 µl	25 µl	25 µl
Thermal cycling profile			
Reverse transcription	30 min 50ºC	--	--
Hot start activation	15 min 95ºC	--	--
Denaturation	--	2 min 94ºC	2 min 94ºC
Cycling	40	35	40
Denaturation	30 sec 94ºC	30 sec 94ºC	30 sec 94ºC
Annealing	1 min 55ºC	1 min 55ºC	1 min 56ºC
Extension	90 sec 72ºC	90 sec 72ºC	1 min 72ºC
Final extension	10 min 72ºC	7 min 72ºC	5 min 72ºC

* included in the 2x kit mastermix; † Included in the 5x kit buffer.

Supplementary Table 2: Primers used in the Pr-RT amplification and sequencing.

Oligo	Use	Direction	HXB2 position	Sequence
RP-1-S	1st PCR/RT-PCR	sense	2016-2041	5′-GAA AAA GGG CTG TTG GAA ATG TGG AA
RP-1-A	1st PCR/RT-PCR	antisense	3685-3716	5′-AAA TTT AGG AGT CTT TCC CCA TAT TAC TAT GC
PR-O-S2b	nested PCR/ sequencing	sense	2080-2107	5′-GCT AAT TTT TTA GGG AAR ATY TGG CCT T
RT-O-A	nested PCR/ sequencing	antisense	3630-3662	5′-TGC CTC TGT TAA TTG TTT TAC ATC ATT AGT GTG
PRsec2A	sequencing	sense	2838-2811	5′-GAT GYG GTA TTC CTA ATT GRA CYT CCC A
RTsec1S	sequencing	antisense	2692-2716	5´-CAA AAA TTG GGC CTGA AAA TCCA TA

Supplementary Table 3: New HIV-1 diagnoses from Basque Country and Galicia (2013-18) included in the study and reported to the HIV National Surveillance System.

		Laboratory		Surveillance*		
		N†	%	N	%	*p*-value
Gender	Male	932	82	1477	81	0.461
	Female	198	18	340	19	
	Unknown & Other‡	28		0		
Transmission route	MSM§	459	54	957	57	0.096
	Heterosexual	345	41	631	37	
	PWID ¶	39	4.6	104	6.1	
	MNSSTꝨ	151		NAʅ		
	Unknown & Other‡	164		125		
Age group	<20	25	2.2	5	1.5	0.131
	20-29	256	23	23	21	
	30-39	396	35	151	33	
	≥40	456	40	280	44	
	Unknown‡	25		0		
Region of origin	Spain	745	70	1259	70	0.881
	Latin America	184	17	301	17	
	Sub-Saharan Africa	85	8.0	159	8.9	
	North Africa	15	1.4	22	1.2	
	Europeð	28	2.6	54	3.0	
	Unknown & Other‡	101		22		
Total	Both Regions	1158	64	1817	100	
	Basque Country	776	86	907	100	
	Galicia	382	42	910	100	

* Data from the Spanish Information Systems on HIV New Diagnoses (SINIVIH); †N: number of patients; ‡ Sexual male, unknown and others categories were not included in the percentage and *p*-value calculations; §MSM: men who have sex with men; ¶PWID: person who injects drugs; ꝨMNSST: Men who have non-specified sexual transmission; ʅNA: Not applicable, MNSST is not a category collected by SINIVIH. ðOther than Spain.

Supplementary Table 4. Factors associated with transmission clusters, univariate/multivariate analysis.

Variables		Univariate analysis		Multivariate analysis	
		OR*	95% CI†	Adjusted OR	95% CI
Gender	Female	Reference		Reference	
	Male	4.0	2.8-5.6	2.1	1.3-3.6
	Transexual	1.7	0.3-11	NA‡	NA
Transmission route	Heterosexual	Reference		Reference	
	MSM§	3.6	2.6-4.9	1.8	1.2-2.6
	MNNSST¶	2.4	1.6-3.5	1.2	0.8-2.1
	PWID⁋	1.1	0.6-2.2	NA	NA
	Other	0.5	0.1-2.2	NA	NA
Region of origin	Spain	Reference		Reference	
	Latin America	0.5	0.3-0.6	0.5	0.3-0.7
	Sub-Saharan Africa	0.06	0.03-0.1	0.2	0.06-0.4
	North Africa	0.4	0.1-1.0	0.5	0.2-1.8
	Europe ʅ	0.4	0.2-0.8	0.5	0.2-1.2
	Other	0.1	0.01-0.9	0.3	0.02-3.0
Age group	<20	1.3	0.3-1.4	NA	NA
	20-29	Reference		Reference	
	30-39	0.6	0.5-0.9	0.6	0.4-0.9
	≥40	0.6	0.5-0.9	0.6	0.4-0.9
Spanish Region	Basque Country	Reference		Reference	
	Galicia	1.9	1.5-2.4	1.2	0.9-1.8
Genetic form	A	0.8	0.4-1.5	NA	NA
	B	Reference		Reference	
	C	0.9	0.5-1.7	NA	NA
	F	2.0	1.2-3.4	2.1	1.1-4.1
	G	0.2	0.08-0.6	0.4	0.1-1.2
	CRF02_AG	0.2	0.1-0.3	0.6	0.3-1.2
	CRF_BF	0.3	0.1-0.3	0.2	0.05-0.5
	URF	0.6	0.4-0.9	0.9	0.5-1.6
	Other	0.2	0.1-0.4	0.3	0.1-0.6

Origin of the patients were not considered in the definition of the TCs. *OR: Odds ratio; †95% CI: 95% confidence interval; ‡NA: Not applicable. Adjusted OR was not calculated for categories with p<0.1 in the univariate analysis; §MSM: Men having sex with men; ¶MNNSST: Men who have non-specified sexual transmission; ⁋PWID: People who inject drugs. ; ʅOther than Spain.

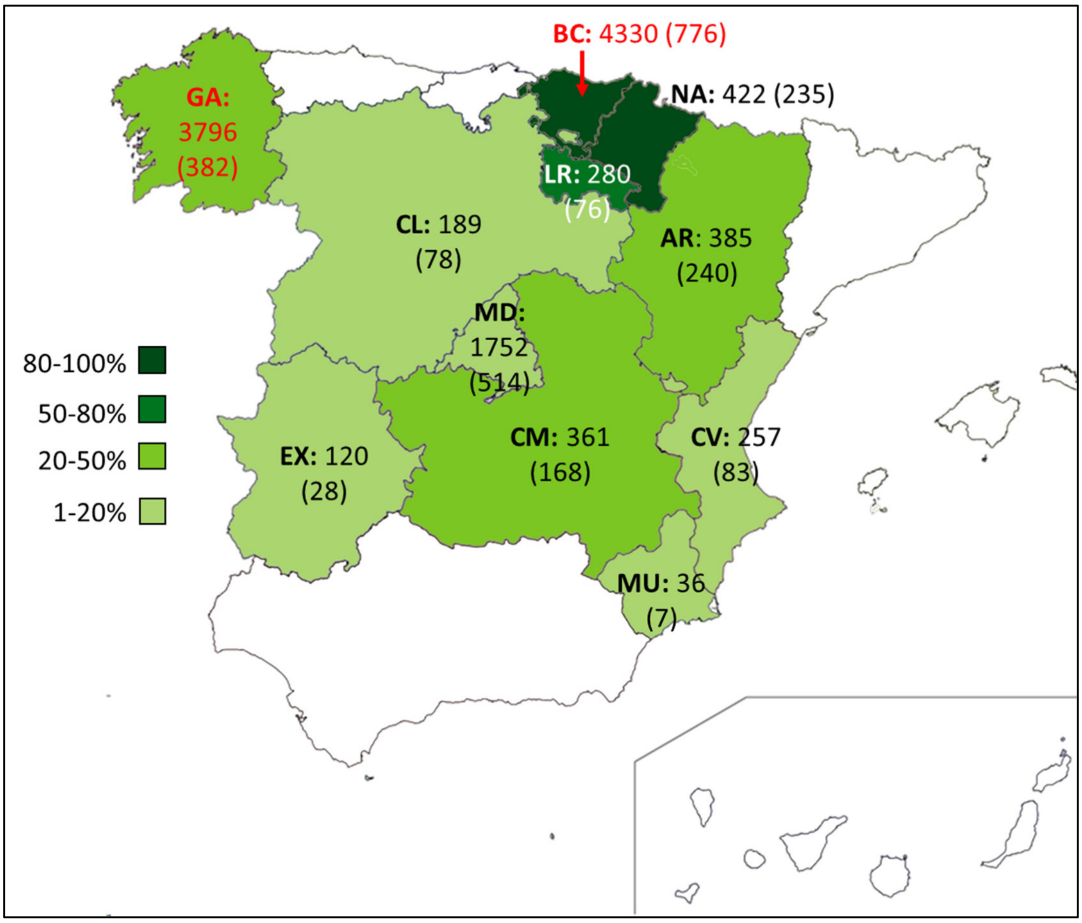

Supplementary Figure 1. Patients with Pr-RT sequences available in our database by region. Total number of patients (samples collected between 1999 and 2019) are indicated in each region. Number of NDs during the studied period (2013-2018) are in parentheses. The green gradient shows the representativeness of the NDs sequenced by us during the studied period (2013-2018) compared with the cases reported to the Spanish information system on new HIV diagnoses (HIV, STI and Hepatitis Surveillance Unit, 2020). Galicia and Basque Country are labeled in red. GA: Galicia; BC: Basque Country; NA: Navarra; LR: La Rioja; AR: Aragón; CL: Castilla y León; MD: Madrid; EX: Extremadura; CM: Castilla La Mancha; CV: Comunitat Valenciana; MU: Murcia.

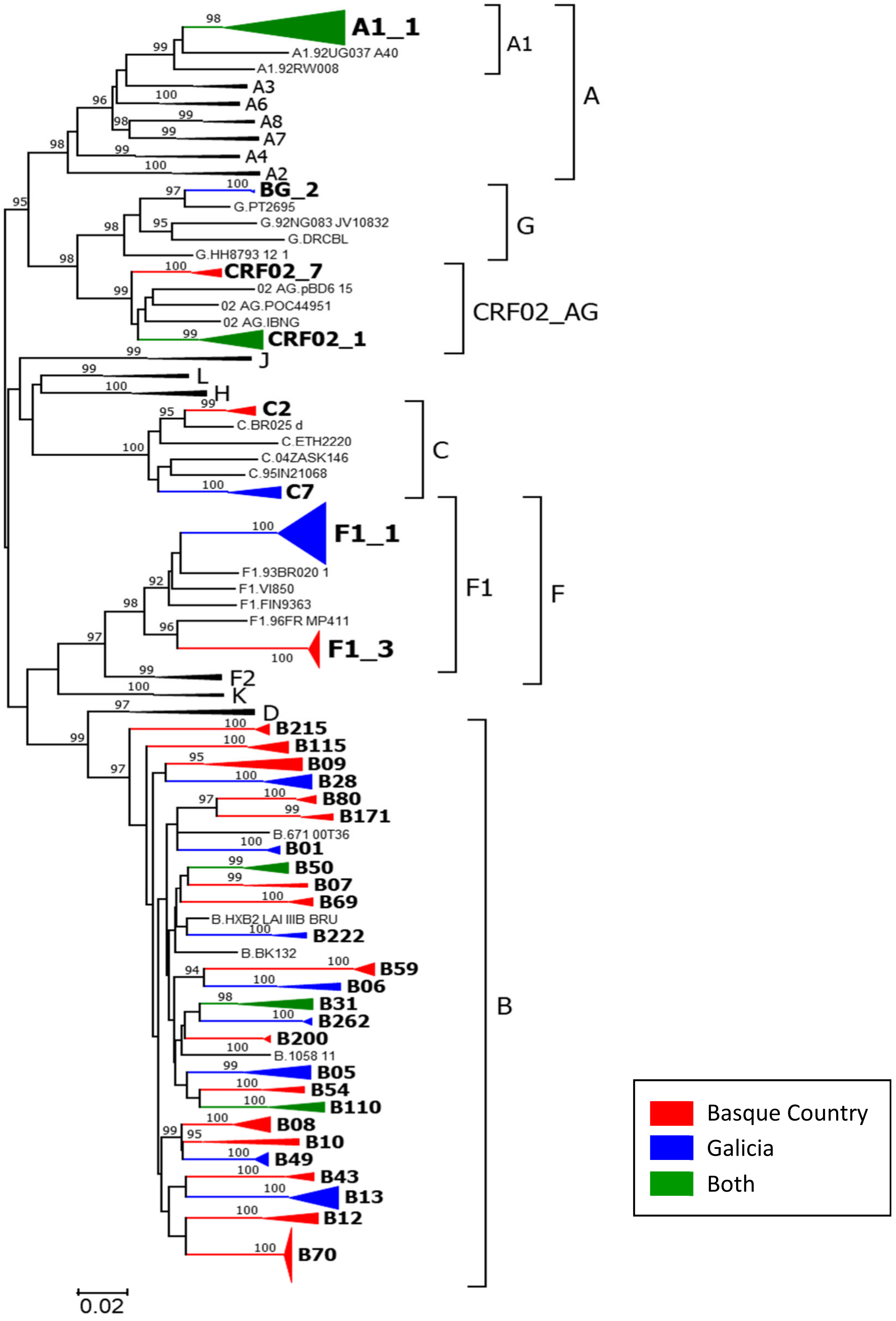

Supplementary Figure 2:

Phylogenetic tree of protease-RT sequences generated with FastTree2. Coloured triangles represent all the TCs ≥ 30 patients and TCs comprising 5 or more patients newly diagnosed in 2013-2018 in Galicia and Basque Country. Only sequences newly diagnosed in the studied period in both regions and subtype references are included. Colours indicate the main region where the TC is circulating.

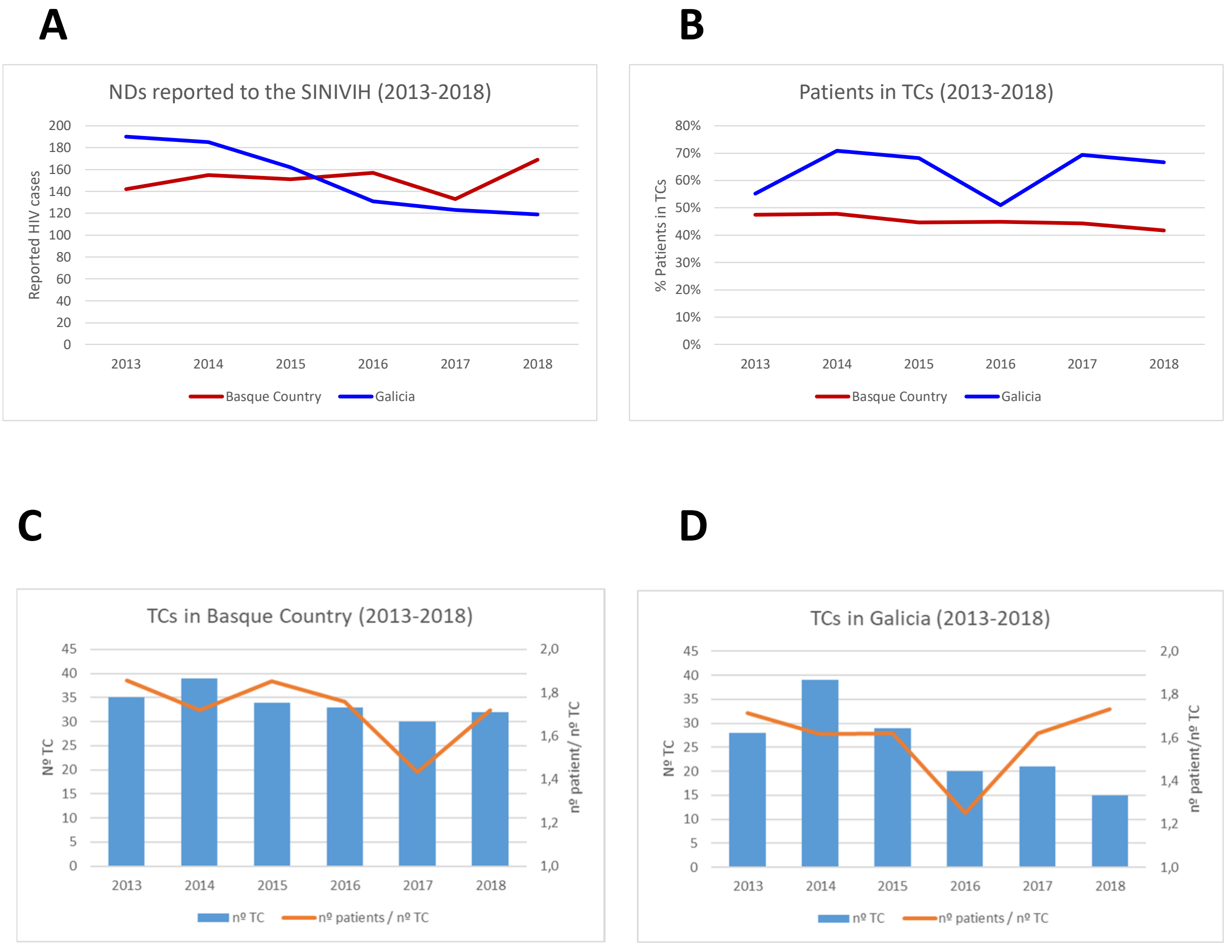

Supplementary Figure 3: Temporal trend of the patients associated to TCs. **Panel A:** NDs reported to the Spanish information system on new HIV diagnoses (SINIVH) in the Basque Country and Galicia during 2013-2018. **Panel B:** Percentage of patients in TCs during the studied period. **Panels C and D**: Number of TCs comprising NDs in the corresponding year and ratio of patients per TC in the Basque Country and Galicia, respectively.
